# Utilization of diverse oligosaccharides for growth by *Bifidobacterium* and *Lactobacillus* species and their in vitro co-cultivation characteristics

**DOI:** 10.1007/s10123-023-00446-x

**Published:** 2023-11-09

**Authors:** Yao Dong, Mei Han, Teng Fei, Huan Liu, Zhonghui Gai

**Affiliations:** 1 Department of Research and Development, Wecare Probiotics Co., Ltd., Wujiang Bridge Road, 1033, Suzhou, 215200 China; 2 Department of Food Science, Shanghai Business School, Shanghai, 200235 China

**Keywords:** *Lactobacillus* group, *Bifidobacterium*, Oligosaccharides

## Abstract

Various approaches have been used to study the relationship between prebiotics and probiotics. The utilization of different carbohydrates by probiotics depends on the biochemical properties of the enzymes and substrates required by the microbial strain. However, few studies have systematically analyzed the ability of probiotics to utilize different prebiotics. Here, we investigated the effects of prebiotics from different manufacturers on the proliferation of 13 strains of the *Lactobacillus* group and the genus *Bifidobacterium* co-cultured in vitro. Inulin, fructose-oligosaccharide (FOS), and galactose-oligosaccharide (GOS) had broad growth-promoting effects. FOS significantly promoted the proliferation of *B. longum*. When strains from *Lactobacillus* group and *Bifidobacterium* were co-cultured, FOS caused each strain to proliferate cooperatively. GOS was effectively used by *L. rhamnosus* and *L. reuteri* for energy and growth promotion. *L. casei* and *L. paracasei* fully metabolized inulin; these strains performed better than other strains from *Lactobacillus* group and *Bifidobacterium*. Media containing a mixture of oligosaccharides had stronger effects on the growth of *B. animalis* subsp. *lactis*, *L. acidophilus*, and *L. rhamnosus* than media containing single oligosaccharides. Thus, different oligosaccharides had different effects on the growth of probiotics, providing a scientific basis for the use of synbiotics in health and related fields.

**Supplementary Information:**

The online version contains supplementary material available at 10.1007/s10123-023-00446-x.

## Introduction

Microbiologically, probiotics are defined as live microorganisms that, when administered in adequate amounts, confer health benefits to the host (Hill et al. 2014). While these probiotics colonize the intestinal and reproductive systems of humans and animals, their metabolites and enzymes also play crucial roles in benefiting the host. Probiotics can improve their hosts’ microecological balance, providing health-related benefits (Swanson et al. 2020b). Probiotics derived from food primarily belong to different microbial species, with *Lactobacillus* group and *Bifidobacterium* being the most common. Zheng et al. (2020) reclassified the genus *Lactobacillus* into 23 groups based on phenotypic, ecological, and genotypic diversity, some of which are *Lactobacillus*, *Lacticaseibacillus*, *Limosilactobacillus*, *Lactiplantibacillus*, *Ligilactobacillus*, and *Latilactobacillus*. Additionally, products containing *Bifidobacterium* species, such as *B. longum* subsp*. longum*, *B. animalis* subsp. *lactis*, *B. breve*, *B. adolescentis*, and *B. bifidum* are commonly found in the market and known for their health benefits (Lugli et al. 2019). *Lactobacillus acidophilus*, *L. plantarum*, and *L. rhamnosus* are also commonly available in the market (Fijan 2014). Some scholars have suggested that certain probiotics can derive enhanced benefits from the presence of prebiotics. Prebiotics can act as a nutritional source for these probiotics, supporting their activity in the intestine. Additionally, when metabolized by probiotics, prebiotics can lead to the production of short-chain fatty acids that further promote probiotic colonization (Swanson et al. 2020b). The International Scientific Association for Probiotics and Prebiotics issued a consensus stating that prebiotics are selectively used by host microorganisms and converted into substances beneficial to host health, thereby updating the definition and scope of prebiotics (Gibson et al. 2017). Prebiotics essentially consist of oligosaccharides such as fructose-oligosaccharide (FOS), galactose-oligosaccharide (GOS), inulin, xylose-oligosaccharide (XOS), stachyose, and resistant dextrin (RD) (Bindels et al. 2015). The combination of probiotics and prebiotics is termed as synbiotics. (Swanson et al. 2020b). These prebiotics possess glycosidic bonds that resist digestion in the small intestine. These compounds, however, can be metabolized by certain probiotics, serving as effective carbon sources that promote their growth and metabolic activity. This ability can give probiotics a competitive advantage in the intestinal tract and in turn, benefit the host (Swanson et al. 2020b; Fuhren et al. 2021). Studies have shown that synbiotic supplementation increases the survival of probiotics as the supplements pass through the digestive tract, allowing the probiotics to effectively colonize the colon (Swanson et al. 2020b). Therefore, synbiotics are often used as dietary supplements to maintain intestinal homeostasis and human microecological health.

The relationship between and colocalization of prebiotics and probiotics has become a recent topic of interest in gut microbial ecology. An increasing number of researchers have conducted studies on this relationship, having explored the physiological function, mechanism of action, and effects of different combinations to understand their basic characteristics and potential application value (Becerra et al. 2015; Rattanaprasert et al. 2019; Zhong et al. 2020). However, few studies have systematically and comprehensively examined the effects of multiple prebiotics on the proliferation of different probiotic species and on synergistic cultures of members from the *Lactobacillus* group and *Bifidobacterium*.

With the background of this knowledge, we sought to explore the growth-promoting effects of 16 prebiotics on selected strains from the *Lactobacillus* group and the genus *Bifidobacterium*. Then, we selected three groups of oligosaccharides with the best growth-promoting effects from then (Bindels et al. 2015) and analyzed the viable count variations in five common probiotics and co-cultures of lactobacilli and bifidobacteria.

## Materials and methods

### Strain information

Thirteen strains were provided by Wecare-bio Probiotics (Suzhou) Co., Ltd.; the details are shown in Table 1. We used five strains of *Bifidobacterium* and eight strains of *Lactobacillus* group (Zheng et al. 2020); they were stored in the China General Microbiological Culture Collection Center. Additionally, to ensure the feasibility of the experiment, we used *Streptococcus thermophilus*, which could not utilize most carbon sources, as a negative control group.

**Table 1:** Strain information involved in the experiment

Genus	Species	Strain	Preservation number
*Bifidobacterium*	*Bifidobacterium animalis* subsp*. lactis*	BLa80	CGMCC No.15410
	*Bifidobacterium longum* subsp*. longum*	BL21	CGMCC No.10452
	*Bifidobacterium bifidum*	BBi32	CGMCC No.16923
	*Bifidobacterium breve*	BBr60	CGMCC No.12915
	*Bifidobacterium adolescentis*	BAC30	CGMCC No.19884
*Lactobacillus*	*Lactobacillus acidophilus*	LA85	CGMCC No.1.12735
*Lacticaseibacillus*	*Lacticaseibacillus rhamnosus*	LRa05	CGMCC. No.1.12734
	*Lacticaseibacillus casei*	LC89	CGMCC No. 15409
	*Lacticaseibacillus paracasei*	LC86	CGMCC No.1.12731
*Limosilactobacillus*	*Limosilactobacillus reuteri*	LR08	CGMCC No. 1.12733
*Lactiplantibacillus*	*Lactiplantibacillus plantarum*	Lp90	CGMCC No.10453
*Ligilactobacillus*	*Ligilactobacillus salivarius*	LS97	CGMCC No.16922
*Latilactobacillus*	*Latilactobacillus sakei*	LSa79	CGMCC No.20125
*Streptococcus*	*Streptococcus thermophilus*	ST81	CGMCC No.15752

All these strains are stored in the China General Microbiological Culture Collection Center (CGMCC)

### Experimental reagents and equipment

#### Prebiotic ingredients information

Sixteen prebiotics (types of inulin, FOS, GOS, XOS, and RD) from different manufacturers were used. Information on their source, number, type, and degree of polymerization (DP) is provided in Supplementary Table S2.

#### Culture media

The culture media used are described in Supplementary Table S3. Following De Man et al. (1960), liquid Man Rogosa Sharpe (MRS) medium was inoculated with the bacterial strains at 2% (vol/vol). For the proliferation media, glucose in liquid MRS medium was replaced with each of the 16 oligosaccharides at 2% (vol/vol). *Bifidobacterium* strains were cultivated in MRS medium containing 0.05% (vol/vol) lin-mupirocin (Qingdao Haibo Biotechnology Co., Ltd.), which was named selective medium 1 (SM1). *L. acidophilus* was grown in MRS medium with lincomycin hydrochloride (Sigma C5269, 2 mg lincomycin hydrochloride diluted in distilled water to 10 mL, added at 0.05% (vol/vol)) and ciprofloxacin (BAYER 02838560, 20 mg ciprofloxacin diluted in distilled water to 10 mL, added at 0.5% (vol/vol)), which was named selective medium 2 (SM2) (International Standards Organisation 2006). Based on the carbon utilization shown in Supplementary Table S1, *L. rhamnosus* or *L. plantarum* was distinguished from *L. acidophilus* using blank MRS medium (without carbon source) with 2% (vol/vol) mannitol as the only carbon source. This was named selective medium 3 (SM3).

#### Main experimental methods

A 722S visible spectrophotometer (Shanghai Lingguang Technology Co., Ltd.) was used to measure the optical density at a wavelength of 600 nm (OD_600_), which is indicative of the turbidity of the fermentation media. A 5-L fermenter (Shanghai Baoxing Biological Equipment Engineering Co., Ltd., BIOTECH-3JG-4) was set up to culture the 13 bacterial strains. A bioreaction online detection system (BODS; Luoyang Huaqing Tianmu Biological Technology Co., Ltd.) was used as a biosensor for automatic online sampling, processing, detection, and sample retention in the bioreactors. Lactic acid production was determined using corresponding enzyme membranes (SBA-40B, Institute of Biology, Shandong Province Academy of Sciences). The membrane, treated with lactate dehydrogenase (LDH), facilitates the enzymatic conversion of lactic acid as it diffuses through. The electrochemical changes resulting from this enzymatic interaction are then captured and translated into accurate lactic acid concentration readings. The number of viable bacteria was determined based on colony-forming units (CFU) and the international standard for food microbiology detection. After co-cultivation of lactobacilli and bifidobacteria, the CFU per milliliter was determined and the individual species were differentiated by plate counting on selective media.

### Experimental design

The 13 bacterial strains were cultured in liquid MRS media at 37 °C for 24 h. The cultures were centrifuged (8000 × g, 10 min) to obtain the bacterial sludge, which were then blended with saline. We then inoculated 1% (wt/vol) bacteria into the 16 proliferation media groups and a liquid MRS medium group (control group); all of them were grown at 37 °C for 24 h. The OD_600_ was determined (after diluting five times) to examine the effects of different oligosaccharide in promoting the growth of the strains. We then selected and tested the three oligosaccharide groups that showed maximum proliferation, as determined using a biosensor.

Using a fermenter and BODS, utilization of the three types of oligosaccharides by the 13 strains was measured. Approximately 1.5 L of liquid MRS medium was prepared as control, and the three experimental oligosaccharide groups were screened and equipped under the same conditions. The OD_600_ and lactic acid production of the 13 strains were monitored. We selected five strains (*B. longum* subsp*. longum* BL21, *B. animalis* subsp. *lactis* BLa80, *L. rhamnosus* LRa05, *L. plantarum* Lp90, and *L. acidophilus* LA85) and measured their populations after growth. The viable bacteria count changes of each strain were calculated. The growth patterns, utilization of oligosaccharides, and lactic acid production of lactobacilli and bifidobacteria in the multi-strain environments were also analyzed after co-cultivation.

### Statistical analysis

All of the fermentation systems were designed with three biological replicates, and each index was determined using three independent samples. All data analyses were performed in R 4.2.0. Using the ggtree package, the relationships between different strains were evaluated by the adjacency method (Yu 2020). The multiple correspondence analysis (MCA) function in the FactoMineR package was used to cluster the acids produced, based on the carbohydrate API 50CH information of the strains (Lê et al. 2008). The OD_600_ values of different strains were presented using the pheatmap package (Galili et al. 2018). The ggplot2 package was used to display the OD_600_, lactic acid production, and viable bacteria counts (Gómez-Rubio 2017).

## Results

### Bioinformatics analysis based on 16 s rRNA and API 50CH information

**Fig. 1:**
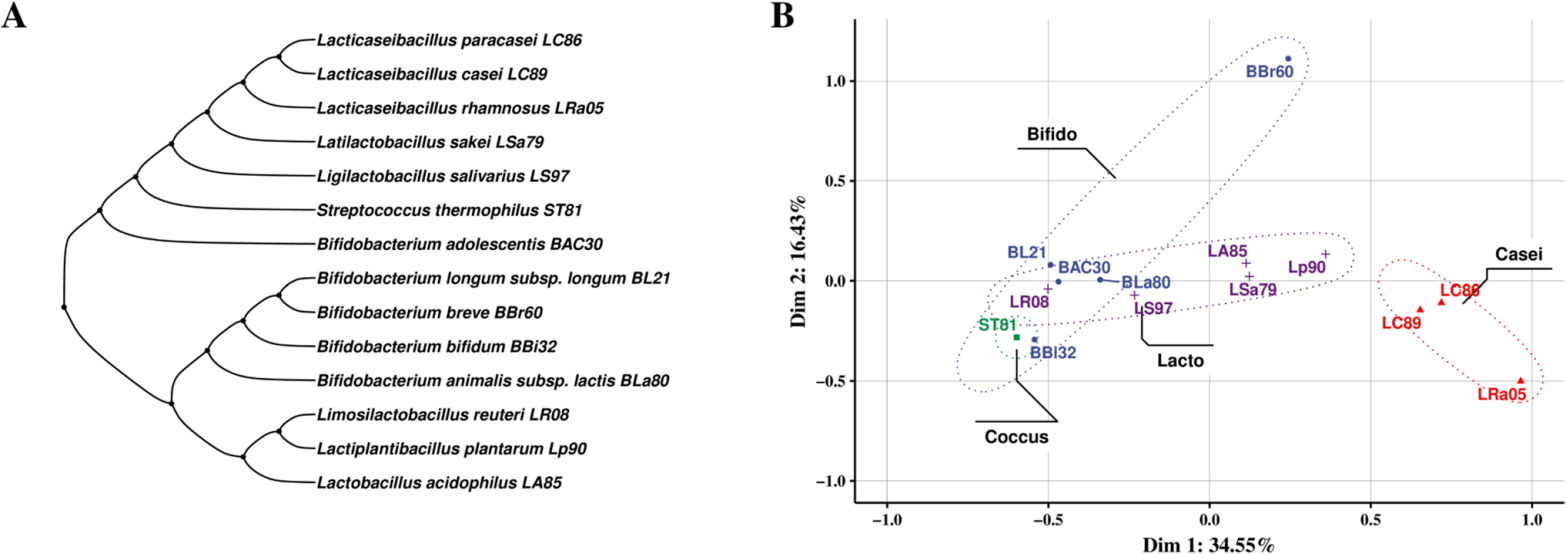
Bioinformatics analysis of the experimental strains. **A** Phylogenetic evolutionary tree of each strain based on 16S rRNA sequence. The ggtree package in R (v4.2.0) was used to evaluate the relationships between strains via the neighbor-joining method. **B** Using multiple correspondence analysis (MCA) and the FactoMineR and MCA functions in R (v4.2.0), we used the API 50CH information of the strains to delineate the relationships between them. We also clustered strains based on carbohydrate utilization

The phylogenetic tree drawn based on 16S rRNA sequence information of 14 strains (including *S. thermophilus* ST81 as a negative control) showed some correlation between the strains at the species level (Fig. 1A). We found that the clusters based on API 50CH information of most of the strains were similar at the species level as well (Fig. 1B).

### Growth of strains from the genus *Bifidobacterium *and the *Lactobacillus* group in media containing different oligosaccharides

**Fig. 2:**
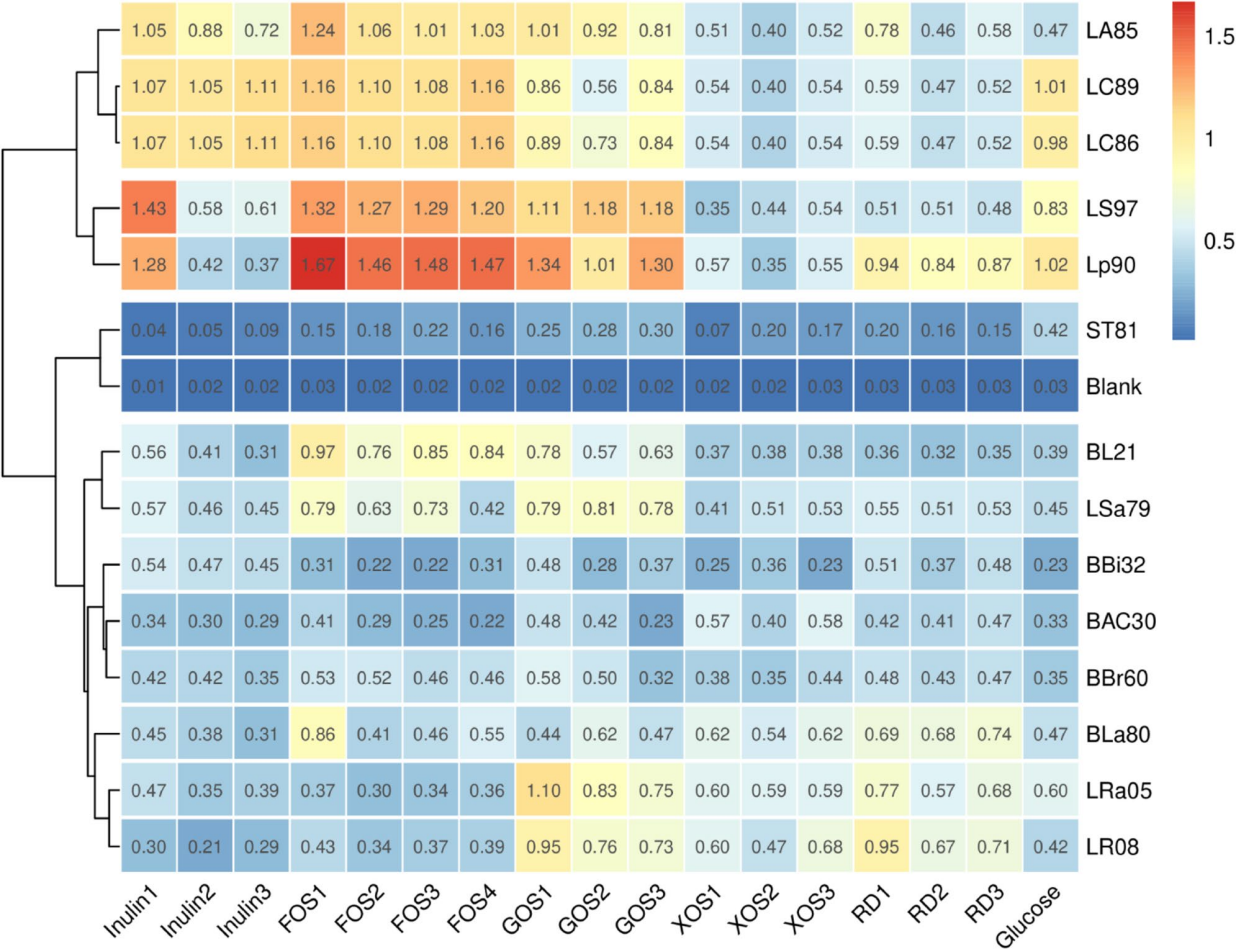
Growth-promoting effects of oligosaccharides on strains from *Bifidobacterium* and the *Lactobacillus* group. The fermentation media of each strain was diluted five times, and the OD_600_ was measured to reflect the change in biomass. In this heatmap, each square represents an OD_600_ value, which was an average of three parallel tests conducted under the same conditions

The non-inoculated medium group was used as the blank control, that is, the OD_600_ of this group was set to 0. Although *S. thermophilus* ST81 was provided glucose, lactose, and sucrose as carbon sources, this strain cannot metabolize most of others (see Table S1). Therefore, *S. thermophilus* ST81 was used as a negative control. The results of blank and negative control confirmed the feasibility of this study (Fig. 2). Among the *Bifidobacterium* species, GOS and FOS proliferation media showed clear growth-promoting effects on the strains BL21, BLa80, and *B. breve* BBr60. The OD_600_ of the BL21 strain cultured with FOS1 was approximately 2.5 times higher (OD_600_ = 0.97) than that of the control strain (OD_600_ = 0.39), and it was slightly stronger than the optical densities observed with the other FOS groups and GOS1. Thus, FOS1 promoted the growth of strain BL21. The OD_600_ with FOS1 was 0.86 for the BLa80 strain, which was 1.8 times higher than that of the control group (OD_600_ = 0.47). RD3 and GOS2 also increased the growth of the BLa80 strain by more than onefold. The growth-promoting effects of GOS1 (OD_600_ = 0.58) and FOS1 (OD_600_ = 0.53) were similar for strain BBr60; both increased growth by approximately 1.5 times over that of the control group (OD_600_ = 0.35). The growth-promoting effects of prebiotics on *B. bifidum* BBi32 and *B. adolescentis* BAC30 differed from those on strains BL21, BLa80, and BBr60. In addition, oligosaccharides made by different manufacturers had different effects; for instance, four types of FOS and three types of GOS showed varying effects on growth. The OD_600_ of the inulin-, RD1-, and GOS1-supplemented BBi32 strain was higher, i.e., more than twice that of the control group. XOS, FOS1, and GOS1 had strong growth-promoting effects on the BAC30 strain, with its OD_600_ being approximately double that of the control group. The ability of *Lactobacillus* group to utilize oligosaccharides reflected strain specificity. The strains Lp90, LS97, LSa79, LC86, and LC89 showed relatively strong growth characteristics in MRS media, but their responses to the prebiotics differed. For example, inulin1, FOS, and GOS promoted growth of the strains Lp90 and LS97, slightly exceeding the effects of glucose. Strains LC86 and LC89 showed similar abilities to utilize oligosaccharides, with their growth efficiently promoted by inulin and FOS. However, they failed to utilize XOS and RD. FOS1 (OD_600_ = 1.24) promoted the growth of the LA85 strain to approximately 2.6 times that of the control group (OD_600_ = 0.47). GOS improved the growth of strains LRa05 and LR08; this was followed by RD1, the effect of which was better than that of glucose. Inulin and FOS had no obvious proliferative effects on the strains LRa05 and LR08. Thus, as shown in Fig. 2, inulin1, FOS1, and GOS1 had strong proliferative effects on certain strains.

### Online monitoring of the growth of *Bifidobacterium* in media containing oligosaccharides

**Fig. 3:**
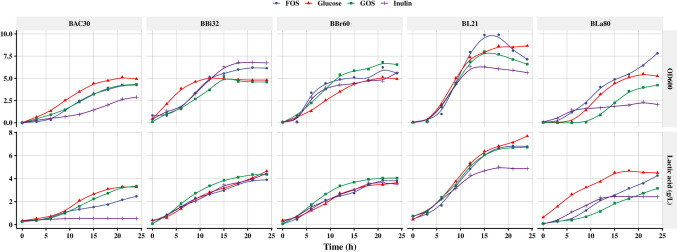
Effects of inulin, fructose-oligosaccharide (FOS), and galactose-oligosaccharide (GOS) on *Bifidobacterium* growth, as monitored online using a bioreaction online detection system (BODS). The effects of inulin, FOS, and GOS on the OD_600_ values and lactic acid production (g/L) of five *Bifidobacterium* strains were monitored online using BODS over 24 h

Based on the preliminary proliferation tests of the 13 stains with various prebiotics (Fig. 2), the three prebiotics with the strongest growth-promoting effects (inulin1, FOS1, and GOS1) were selected as experimental groups for further study. Using a 3-L fermenter and BODS, the effect of these three prebiotics in promoting growth and lactic acid production by the five *Bifidobacterium* strains and eight strains from the *Lactobacillus* group was monitored online for 24 h. The OD_600_ value was correlated with lactic acid production, that is, growth of the strain affected lactic acid production (Fig. 3). The BL21 strain showed a strong ability to utilize each prebiotic; its lactic acid yield (> 5 g/L) was higher than that of the other four *Bifidobacterium* strains. None of the prebiotics affected the lag phase of growth of the five *Bifidobacterium* strains (Fig. 3), which was approximately 3–5 h. However, they did change the logarithmic and stationary phases of growth of the strains. For example, FOS accelerated the growth rate of strains BL21 and BLa80 in logarithmic phase and prolonged the time for them to enter the stable phase, thus making the strains grow better. Lactic acid production was also affected similarly by all three prebiotics, with a tendency towards growth curves correspondingly. FOS improved the proliferation of the different *Bifidobacterium* strains; the effects were comparable or even superior to the control groups composed of the strains BL21 and BBr60 and the GOS-supplementation group of BLa80. The logarithmic growth phases of the inulin-supplemented strains BAC30 and BLa80 were relatively short, while the strains quickly showed signs of stationary phase.

### Online monitoring of the growth of strains from the *Lactobacillus* group in the media containing oligosaccharides

**Fig. 4:**
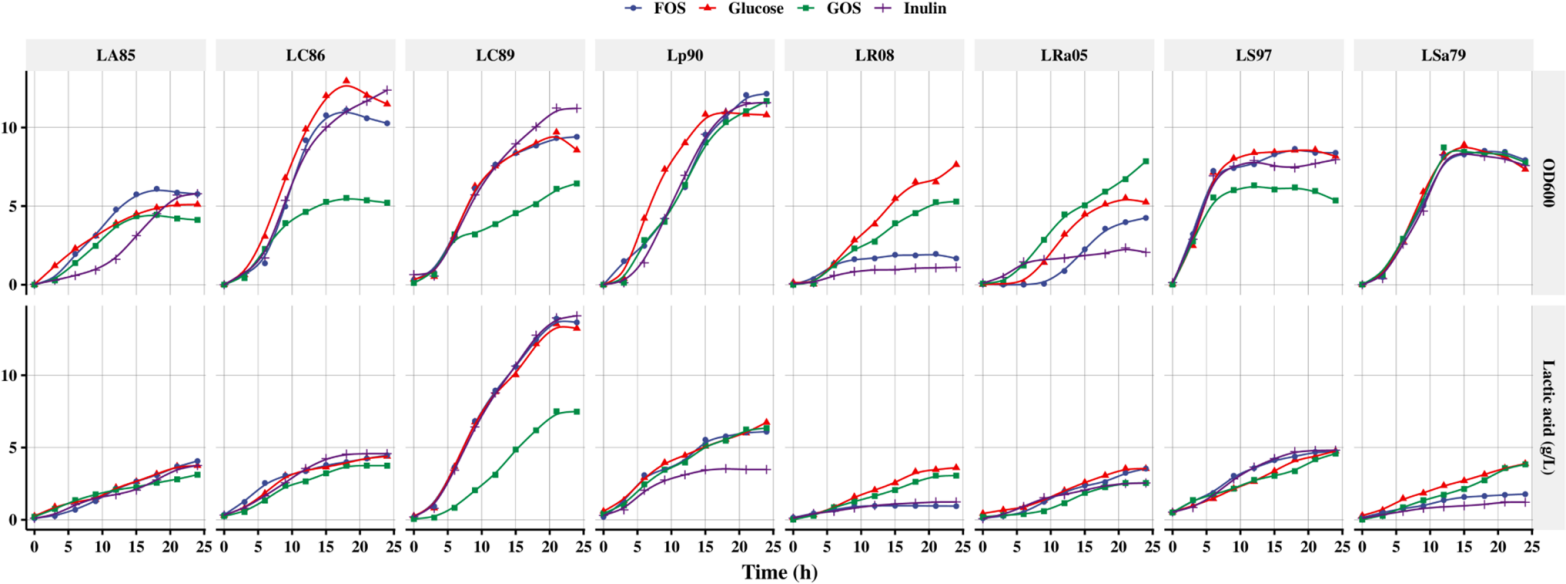
Effects of inulin, fructose-oligosaccharide (FOS), and galactose-oligosaccharide (GOS) on the growth of lactobacilli, as monitored online using a bioreaction online detection system (BODS). The effects of inulin, FOS, and GOS on the OD_600_ value and lactic acid production (g/L) of eight strains from *Lactobacillus* group were monitored online using BODS over 24 h

As shown in Fig. 4, inulin and FOS were efficiently utilized by most of the strains from the *Lactobacillus* group, with the exception of LR08 and LRa05. The growth initiation phases of the experimental and control groups of the other strains from the *Lactobacillus* group were similar (approximately 3–5 h), except for those of LR08 and LRa05. The OD_600_ values of the LC89, LC86, and Lp90 strains were higher than those of the other lactobacilli. The growth trends of the strains LC89 and LC86 were similar, both showing a below-average utilization of GOS. In addition, the LC89 strain showed higher lactic acid production (except when grown with GOS; other three groups showed > 10 g/L lactic acid) than the other strains (around 5 g/L); all of the experimental groups showed similar growth trends. Although the LC86 strain showed good proliferation with all of the prebiotics, it showed little improvement in acid production; there was also little difference across the experimental groups. The utilization of inulin, FOS, and GOS by the LA85, Lp90, LS97, and LSa79 strains was similar, with all of the strains effectively metabolizing the three prebiotics for growth. However, inulin did not efficiently promote lactic acid production by the Lp90 and LSa79 strains; its effect was inferior to that of the other prebiotics. The growth adaptation period of the LA85 strain in the inulin-supplemented group was longer, but its growth and utilization ability in the logarithmic phase was better than in the other groups. The OD_600_ value of the LA85 strain in the stable phase was thus close to that of other strains. The effects on LR08 and LRa05 were slightly different. GOS had a stronger growth-promoting effect on these strains, with its proliferation effect on the LRa05 strain being higher than that of glucose. FOS and inulin supplementation inhibited the growth of and lactic acid production by the LR08 strain.

### Effects of inulin, FOS, and GOS on the populations of *Bifidobacterium* and *Lactobacillus* group

**Fig. 5:**
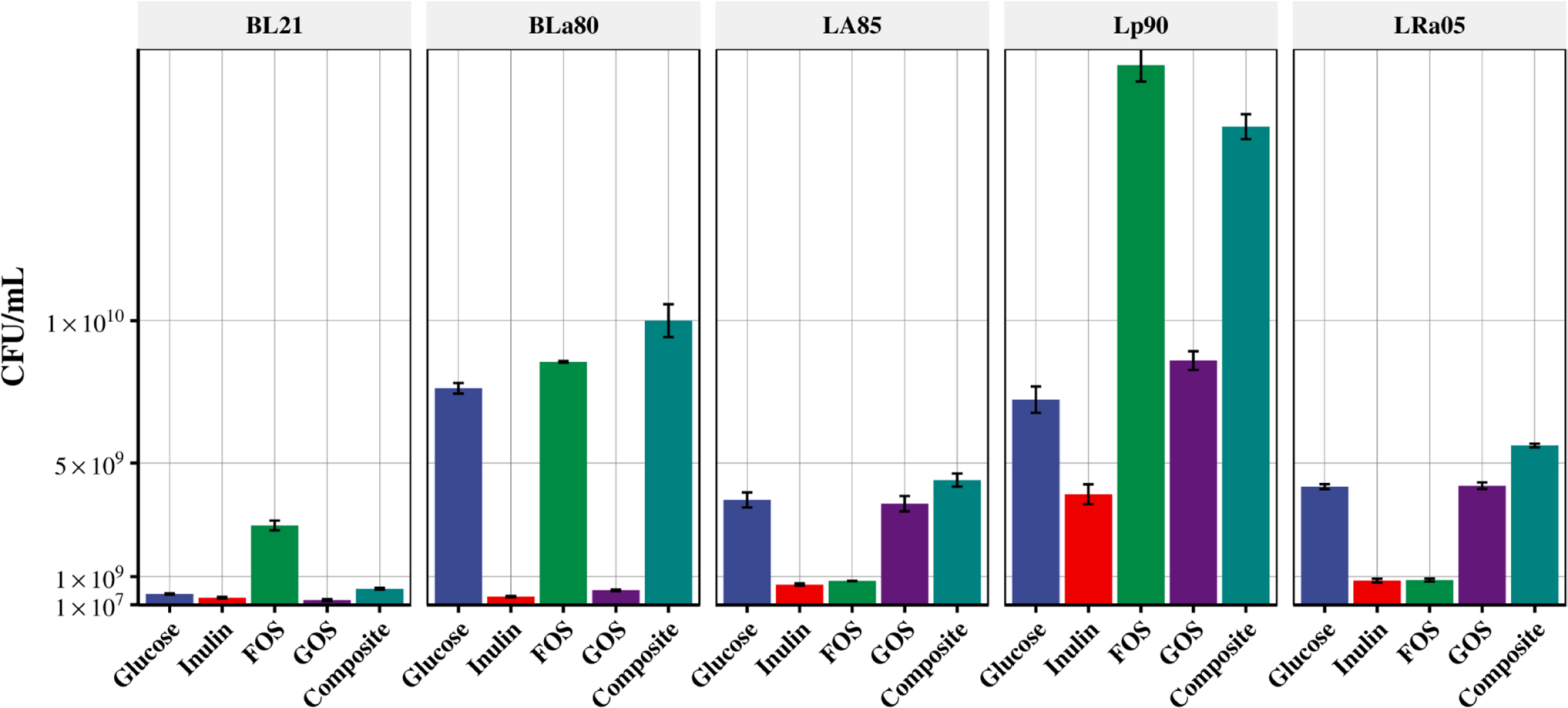
Effects of oligosaccharides on populations of *Bifidobacterium* and lactobacilli. MRS medium was used as the control group, and inulin, fructose-oligosaccharide (FOS), galactose-oligosaccharide (GOS), and composite medium were used as the experimental groups. The changes in the populations of each strain (*B. longum* subsp*. longum* BL21, *B. animalis* subsp*. lactis* BLa80, *L. acidophilus* LA85, *L. plantarum* Lp90, and *L. rhamnosus* LRa05) after 24 h of growth were recorded. The composite medium consisted of the three oligosaccharides—inulin, FOS, and GOS—at a ratio of 1:1:1

Based on the online BODS monitoring results, strains of *Lactobacillus* group and *Bifidobacterium* commonly used in the market were selected: LA85, Lp90, LRa05, BL21, and BLa80. To explore the effects of oligosaccharides on the growth of probiotics, the populations of these five strains were examined (Fig. 5). The preference of different probiotics for specific oligosaccharides also affected the viable counts of the strains. The BL21 strain showed strong utilization of FOS. The viable count also significantly increased to about seven times that of the control group as this strain grew. This indicated that the BL21 strain could use FOS better than glucose. The population of the BL21 strain increased in the composite group, but not as effectively in the group with FOS alone. Inulin and GOS alone had weak effects on the population of strain BLa80, whereas FOS alone had a stronger effect than the control group. When the three oligosaccharides were mixed, the number of viable bacteria (composite group) was about double that of the FOS group, indicating that the composite medium improved the utilization of the three prebiotics by strain BLa80. The same was observed for the strains LA85 and LRa05. Under the effects of inulin and FOS alone, the viable counts of strains LA85 and LRa05 were low, indicating reduced growth compared to a normal glucose culture medium. Only the viable counts of the GOS groups were close to that of the control group. However, the respective population of LA85 and LRa05 increased significantly, even exceeding that of the control composite group, when a mixture of inulin, FOS, and GOS was provided as a carbon source. The Lp90 strain showed strong growth ability, with the viable count of the FOS group being higher than of the GOS, inulin, and control groups (more than 1 billion in all groups). In the composite group, the Lp90 strain population exceeded 10 billion, which was about twice that in glucose. However, its viable count was slightly lower than in the FOS group.

### Effects of oligosaccharides on the populations of co-cultures of strains LA85, Lp90, and BLa80

**Fig. 6:**
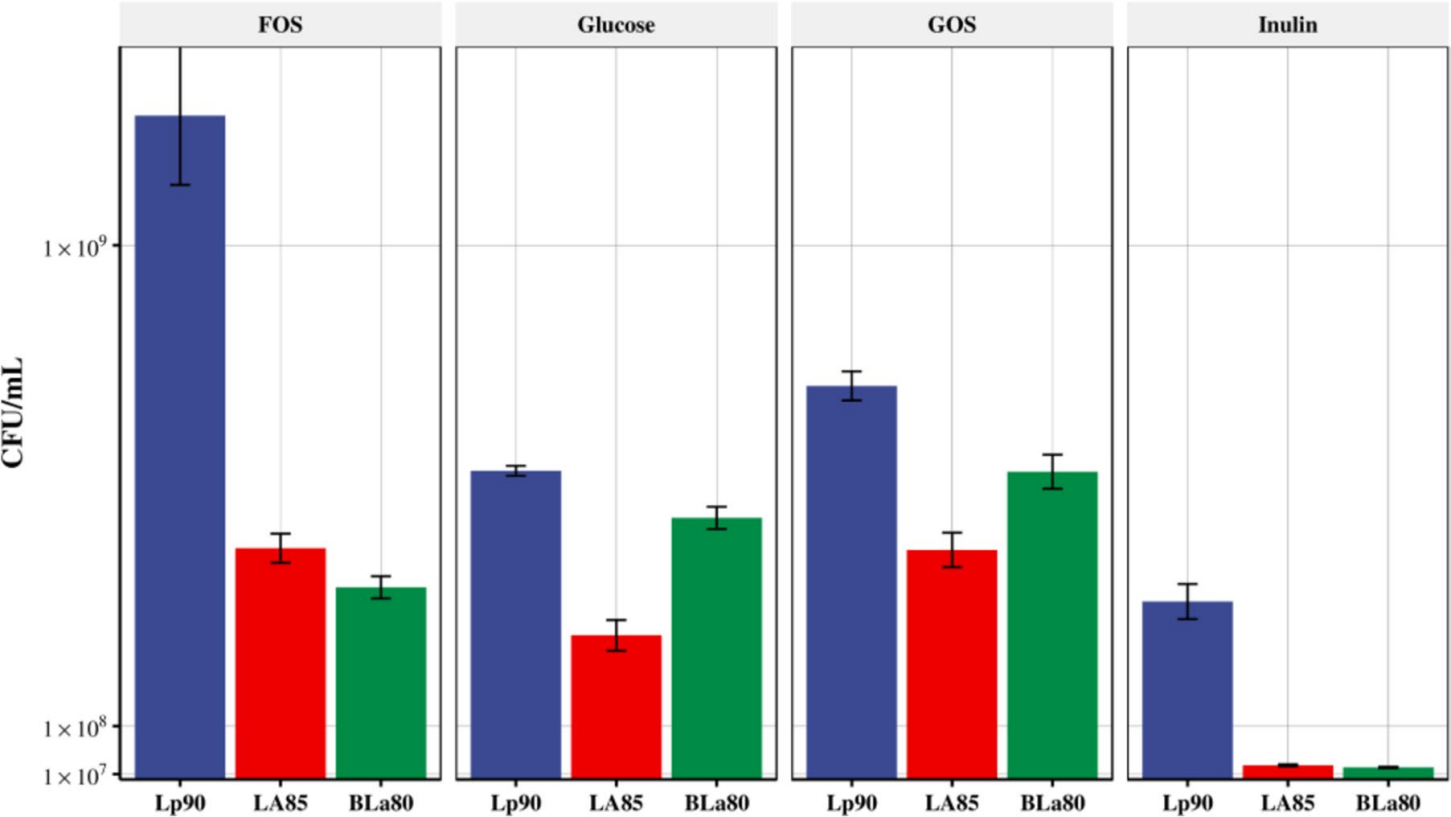
Effects of oligosaccharides on populations of *Bifidobacterium animalis* subsp*. lactis* BLa80, *Lactobacillus acidophilus* LA85, and *L. plantarum* Lp90 in a co-culture. MRS medium was used as the control group, and inulin, fructose-oligosaccharide (FOS), and galactose-oligosaccharide (GOS) were used as the experimental groups. The changes in the populations of each strain after 24 h of growth were recorded. The populations of the strains Lp90, LA85, and BLa80 were determined by plate counting with selective media. The strains LA85, Lp90, and BLa80 were detected using the selective media SM2, SM1, and SM3, respectively

**Fig. 7:**
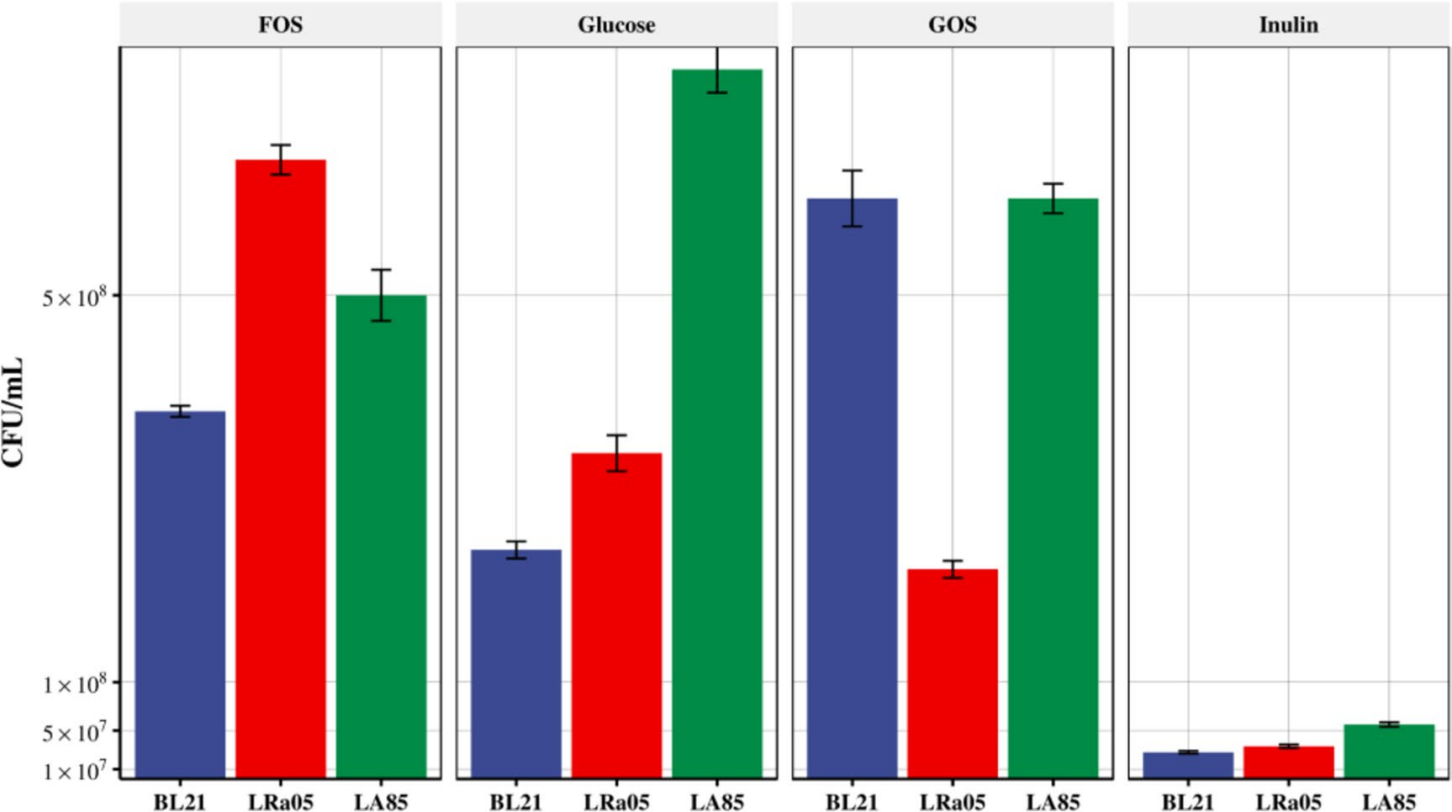
Effects of oligosaccharides on populations of *Bifidobacterium longum* subsp*. longum* BL21, *Lactobacillus acidophilus* LA85, and *L. rhamnosus* LRa05 in a co-culture. MRS medium was used as the control group, and inulin, fructose-oligosaccharide (FOS), and galactose-oligosaccharide (GOS) were used as the experimental groups. The changes in the populations of each strain after 24 h of growth were recorded. The populations of the strains BL21, LA85, and LRa05 were determined by plate counting with selective media. The strains LA85, LRa05, and BL21 were detected using the selective media SM2, SM1, and SM3, respectively

After analyzing the effects of oligosaccharides on the population of the five common probiotics, their effects on the *Lactobacillus* group and *Bifidobacterium* co-cultures were investigated. Considering the number of living bacteria detected and commonly available synbiotics in the market, we set the strains LA85, Lp90, and BLa80 as one combination (Fig. 6) and LA85, LRa05, and BL21 as the other (Fig. 7). As shown in Fig. 6, compared with the control, the growth and populations of these strains increased when they were co-cultured in GOS. In the FOS co-culture, the Lp90 strain showed a competitive advantage, and the proliferation of the BLa80 strain was inhibited (viable count decreased compared with the control). The same was observed in the inulin groups, with the viable count of each strain limited in the co-culture. As shown in Fig. 7, compared with the control group, the growth of strain BL21 significantly improved when co-cultured in GOS, although the populations of the other two strains were slightly reduced. The viable count of strain BL21 doubled more than twice, indicating a competitive advantage. When the three strains were co-cultured with FOS, the LRa05 strain showed a competitive advantage. Its viable count level nearly doubled and that of strain BL21 also significantly increased. However, inulin inhibited the growth of each strain in the co-culture, indicating that it may not be suitable for such cultures.

## Discussion

In this paper, we show that different species of probiotics have different preferences for sources of oligosaccharides. We explored the growth-promoting effects of inulin, FOS, GOS, XOS, and RD from different manufacturers when used as the sole carbohydrate sources for in vitro probiotic growth. Importantly, we speculated that the growth-promoting effects were related to the characteristics of different oligosaccharides and the physiological and biochemical characteristics of different probiotics. We screened three groups of prebiotics with good value-added effects, namely inulin (Vilof), FOS (Quantum Hi-Tech, QTH), and GOS (QTH), for further study. The OD_600_ value and lactic acid production of the bacterial strains were monitored online using BODS. The population variations and viable counts of each strain grown as co-cultures in media containing different prebiotics reflected the potential scientific compatibility of oligosaccharides and probiotics. A given oligosaccharide had different growth-promoting effects on each strain, possibly due to the growth characteristics of the strains and the abilities of the enzymes to hydrolyze different carbon sources (Valdés-Varela et al. 2017).

Most *Bifidobacterium* strains, especially *B. longum* subsp*. longum* BL21, showed strong utilization of FOS and GOS. Its growth, population, and lactic acid production were better in the FOS-supplemented group than those of the other *Bifidobacterium* species and even some lactobacilli. This might align with the concept of synergistic synbiotics (Swanson et al. 2020a), where the probiotic and prebiotic are chosen to interact, maximizing the attributes of the strain. The unique enzymatic capability of the strain allows it to hydrolyze these oligosaccharides and thrive on them, emphasizing this synergistic interaction. Furthermore, BL21’s proliferation ability in FOS was also higher than in ordinary glucose cultures. This may be because the BL21 strain can produce enzymes that hydrolyze FOS (Valdés-Varela et al. 2017). Studies have also reported that the BL21 strain can naturally transport and degrade FOS, GOS, and other oligosaccharides, effectively decomposing them, secreting more related enzymes, and producing nutrients for growth (Margolles and de los Reyes-Gavilán, 2003; Liu et al. 2011; Pokusaeva et al. 2011; Parhi et al. 2022). These processes produce large amounts of acid, effectively reducing the pH value but also increasing the population. In co-cultures, we found that the population of strain BL21 cultured in GOS exceeded that in FOS, although online monitoring of OD_600_ showed that FOS was more conducive to its growth. This may be because the strain produces a glycosidic hydrolase that can act on GOS. GOS utilization increased when the BL21 strain was mixed with lactobacilli, which, in turn, increased its growth and viable count (Macfarlane et al. 2008; Watson et al. 2013; Wilson and Whelan 2017). Related studies (Wilson and Whelan 2017; Fuhren et al. 2021) have shown that GOS is fully utilized by this strain, which significantly increases proliferation and exopolysaccharide (EPS) production. EPS is a capsular or mucous polysaccharide that can adhere to or reside on the cell surface, trigger immune and anti-tumor responses in the host, and improve colonization, which is closely related to population levels. The BL21 strain grown in GOS media showed a competitive advantage and significant increase in population. The ability of the BBi32 strain to utilize inulin (Vilof, GFn, G = glucose, F = fructose) was stronger than its ability to utilize FOS and GOS. Although this may reflect an experimental error, some studies have shown that inulin and FOS had clear growth-promoting effects on *B. bifidum*; this may be related to the chain length and structure of inulin (Kelly 2008; Martinez et al. 2015; Thum et al. 2015). Muramatsu et al. identified a glycosidase called fructo-furanosidase (β-FFases, EC 3.2.1.26), which was capable of degrading fructans in *Bifidobacterium* and belonged to the glycosidase family GH32 (Muramatsu et al. 1993). Inulin is a fructan mixture composed of D-furanosaccharide molecules linked by β (2 → 1) glycosidic bonds, usually with a DP of 2–60. When the DP is low, these molecules are called short-chain FOS. As *B. bifidum* may produce inulinase in the presence of inulin, an environment full of small-molecule oligosaccharides may facilitate their fast metabolism (Sanders et al. 2019).

Different species of the *Lactobacillus* group showed vast differences in their preferences for oligosaccharides. The inulin utilization abilities of strains LC89 and LC86 were the best among the 13 analyzed species. Their API 50CH data were positive for the use of inulin, indicating that they could metabolize different oligosaccharides and produce inulinase to degrade inulin polysaccharide into monosaccharides for glycolysis. Analogously, *L. paracasei* showed different FOS metabolism patterns. This species has been shown to utilize long-chain inulin and express extracellular inulinase, which is anchored on the outer cell wall and degrades inulin into glucose and fructose. These are then transported within the cell for further metabolism (Boger et al. 2018; Sanders et al. 2019). Although the API 50CH data showed that the strains LA85, Lp90, and LS97 could not hydrolyze inulin, they did indeed utilize inulin (Vilof) well. This may be because of the structure of inulin and transport mechanisms of different strains. Researchers have speculated that the transportation systems and inulinases of most strains from *Lactobacillus* group, such as NCFM and WCSF1, can act on inulin and fructan as they do on FOS; this is likely because the inulinase resides in the cytoplasm (Saulnier et al. 2007; Andersen et al. 2012). FOS or inulin (DP 3–5) are transported into the cell and then hydrolyzed by the intracellular glucosidase or inulinase into monosaccharides, which may be further hydrolyzed and enter glycolysis (Saulnier et al. 2007; Andersen et al. 2011). This pattern has been observed in *L. plantarum*, *L. acidophilus*, *L. salivary*, and most *Bifidobacterium* species (Andersen et al. 2011; Wang et al. 2020; Hussain et al. 2021).

*L. reuteri* and *L. rhamnosus* showed a high level of utilization of GOS as they secreted enzymes that decomposed GOS into basic nutrients, which, in turn, promoted metabolism and bacterial reproduction. Studies have shown that in the lactose operon in *L. reuteri*, the *lac* gene can encode hydrolases, transporters, or galactoside substrates essential for GOS metabolism. This enables the cell to fully utilize GOS (Rattanaprasert et al. 2019; Hussain et al. 2021). In addition, in the two experimental co-cultures of *Bifidobacterium* and lactobacilli, *L. plantarum* Lp90 and *L. rhamnosus* LRa05 showed competitive advantages in their respective systems. This may be related to FOS metabolism. Most species within the lactobacilli group metabolize FOS through a specific pathway, but Buntin et al. reported that *L. plantarum* has a fosRABCDXE operon (fructan β-glucosidase) in addition to the pts1BCA operon (FOS and inulin metabolism) (Buntin et al. 2017). This implies that *L. plantarum* can hydrolyze FOS through two pathways, both extracellularly and intracellularly, thereby accelerating FOS utilization (Kaplan and Hutkins 2003; Wang et al. 2020) and offering a competitive advantage. All of the strains grown in the whole FOS fermentation culture system proliferated cooperatively, indicating a beneficial status.

Our study provides a theoretical basis for the compatibility of different prebiotics and probiotics and may facilitate the application of synbiotics. We used only some representative oligosaccharides and common *Bifidobacterium* and strains from *Lactobacillus* group for our experiments. These combinations were chosen for their significance in scientifically proving the compatibility of strain-specific oligosaccharides as prebiotics and probiotics. However, due to the diversity of oligosaccharide species, composition, and structure, and of the number of probiotics species, we could not accurately test and predict the interactions of all existing oligosaccharides and probiotics.

## Conclusion

In this study, the effects of oligosaccharides in promoting growth and lactate production, and populations of different probiotic strains were analyzed, which could provide an exploration into the relationship between these diverse genera strains and prebiotics, shedding light on potential trends. The preferences of different probiotics, which showed similar trends in proliferation and lactic acid production, for specific oligosaccharides were compared. Different types of oligosaccharides are metabolized through distinct pathways and are utilized to different extents. To provide a scientific basis for synbiotic product distribution and to optimize the use of probiotics, it is necessary to conduct in-depth studies on metabolic pathways and metabolites that stimulate probiotics and thus improve their function.

### Supplementary Information

Below is the link to the electronic supplementary material.

Supplementary file1 (XLSX 21 KB)

## Data Availability

The data and materials used in this study are available upon request from the corresponding author. Please contact zhgai@aliyun.com for inquiries regarding data availability.
